# The CB1R rs2023239 receptor gene variant significantly affects the reinforcing effects of nicotine, but not cue reactivity, in human smokers

**DOI:** 10.1002/brb3.1982

**Published:** 2020-12-25

**Authors:** Chidera C. Chukwueke, William J. Kowalczyk, Marie Gendy, Richard Taylor, Rachel F. Tyndale, Bernard Le Foll, Stephen J. Heishman

**Affiliations:** ^1^ Translational Addiction Research Laboratory Centre for Addiction and Mental Health (CAMH) Toronto ON Canada; ^2^ Department of Pharmacology University of Toronto Toronto ON Canada; ^3^ Intramural Research Program National Institute on Drug Abuse Baltimore MD USA; ^4^ Department of Psychology Hartwick College Oneonta NY USA; ^5^ CAMH Campbell Family Mental Health Research Institute Toronto ON Canada; ^6^ Division of Brain and Therapeutics Department of Psychiatry University of Toronto Toronto ON Canada; ^7^ Acute Care Program CAMH Toronto ON Canada; ^8^ Department of Family and Community Medicine University of Toronto Toronto ON Canada; ^9^ Institute of Medical Sciences University of Toronto Toronto ON Canada; ^10^ Centre for Addiction and Mental Health Institute for Mental Health Policy Research Toronto ON Canada

**Keywords:** CB1R, genotype, nicotine cue reactivity, nicotine dependence, nicotine reinforcement

## Abstract

**Introduction:**

The cannabinoid CB1 receptor (CB1R) has been shown in preclinical studies to be involved in nicotine reinforcement and relapse‐like behavior. The common single nucleotide polymorphism (SNP) rs2023239 may code for an alternative CB1R protein, alter CB1R expression, and be involved in nicotine dependence. To date, no study has explored the relationship between this SNP in CB1R and specific phenotypes of nicotine dependence.

**Methods:**

The current study investigated the influence of CB1R rs2023239 in nicotine reinforcement and craving in regular cigarette smokers. Current smokers (*n* = 104, cigarettes per day ≥ 10) were genetically grouped (C allele group vs. No C allele group) and underwent laboratory measures of nicotine reinforcement and smoking cue‐elicited craving. Nicotine reinforcement was assessed using a forced choice paradigm, while a cue‐reactivity procedure measured cue‐elicited craving.

**Results:**

These results show that smokers with the C allele variant (CC + CT genotypes) experienced a lower nicotine reinforcement effect compared to those without the C allele (TT genotype). These results were similar in both our subjective and behavioral reinforcement measures, though the subjective effects did not withstand controlling for race. There was no difference between genotype groups with respect to cue‐elicited craving, suggesting a lack of influence in cue reactivity.

**Conclusion:**

Taken together, these results suggest that the variation in the CB1R (i.e., rs2023239 SNP) may play a larger role in nicotine reinforcement compared to cue reactivity. This work provides impetus to further understand the physiological mechanisms that explain how CB1Rs influence nicotine dependence phenotypes.

## Graphical abstract

This study investigates the effect of a CB1R single nucleotide polymorphism (rs2023239) on smoking phenotypes. The results show that smokers with the C allele variant (CC + CT genotypes) experienced a lower nicotine reinforcement effect compared to those without the C allele (TT genotype). This polymorphism does not seem to affect smoking cue reactivity.

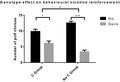

## 1 INTRODUCTION

Tobacco smoking is the leading cause of preventable death (Warren et al., 2014), yet quitting smoking is only achieved by about one‐third of those who try (Babb et al., 2017). This suggests a deficit in the effectiveness of available treatment options in nicotine dependence. Improvements to smoking cessation treatment may come from capitalizing on genetic variation in order to personalize treatments (Bierut et al., 2014).

The cannabinoid system has been shown to play a role in nicotine dependence. This system includes the cannabinoid CB1 and CB2 receptors (CB1R, CB2R), as well as endogenous cannabinoids, and the processes involved in their biosynthesis and transmitter function. There is evidence to support a role of CB1R in nicotine‐related processes. Preclinical studies have showed that blockade of the CB1R, via selective inverse agonist Rimonabant (SR141716), decreases nicotine self‐administration (Cohen et al., 2002) and conditioned place preference (Le Foll & Goldberg, 2004), which are models of nicotine reinforcement and reward. Rimonabant also decreased the reinstatement of previously extinguished nicotine‐seeking in rats (Cohen et al., 2005; Diergaarde et al., 2008; Forget et al., 2009), which can be viewed as relapse‐like behavior. In contrast, CB1R stimulation via a CB1/2R agonist (WIN 55,212‐2) has been shown to increase nicotine self‐administration under progressive ratio schedule of reinforcement and to produce nicotine‐seeking in a drug‐induced reinstatement paradigm (Gamaleddin et al., 2012). Taken together, this suggests a critical role of CB1R in both nicotine reinforcement and relapse‐like behavior in animal models.

The CB1R rs2023239 is a common single nucleotide polymorphism (SNP) present in the CB1R encoding gene. This SNP results in T to C substitution in intron 2 of the gene which may affect CB1R transcription (Zhang et al., 2004). While the mechanistic impact of the rs2023239 CB1R variant is not well characterized, a postmortem human study associated the G allele (complementary to the rs2023239 C allele on the forward genomic DNA strand) of this SNP, along with 2 other associated SNPs in a TAG haplotype (i.e., base “T” at rs806379, “A” at rs1535255, and “G” at rs2023239), with altered mRNA expression (Zhang et al., 2004). Another human postmortem study showed an association between the rs2023239 C allele and increased CB1R expression in the prefrontal cortex (Hutchison et al., 2008). These postmortem results have been corroborated by an in vivo studies showing increased CB1R ligand binding (Hirvonen et al., 2013) and increased CB1R density in human peripheral lymphocytes (Ketcherside et al., 2017) in individuals with the C allele of the rs2023239 SNP, suggesting an increase in CB1R expression.

The rs2023239 SNP, and its related TAG haplotype, have been associated with substance dependence (Chen et al., 2008; Zhang et al., 2004). Of particular note is the association between the rs2023239 and smoking initiation, as well as nicotine dependence (Chen et al., 2008). This evidence indicates that this functional SNP may be involved in aspects of nicotine dependence. These postmortem, in vivo binding data, and association data suggest a functional role of rs2023239 (or a variant in high linkage disequilibrium) that warrants further study. To date, the relationship between this SNP and specific strong endophenotypes of nicotine dependence has not been explored.

The current study explored the effect of the CB1R rs2023239 variant on human laboratory measures of nicotine dependence endophenotypes. Specifically, we examined the effect of rs2023239 variants on nicotine reinforcement and craving, measured by a forced choice and cue‐reactivity paradigm respectively. We hypothesized that the CC + CT (C group) genotype of the rs2023239 SNP, putatively associated with greater CB1R expression, would result in greater nicotine reinforcement and smoking cue reactivity. Additionally, sex differences have been found in various aspects of smoking (Cosgrove et al., 2014; Doran, 2014). Therefore, we secondarily explored the effect of sex on the interaction between genotype and our laboratory measures of smoking phenotypes.

## 2 MATERIALS AND METHODS

### 2.1 Participants

The collection of the data reported here has been previously described (Chukwueke et al., 2020). Briefly, this analysis was done on a data set collected in three separate studies. The first study investigated the nicotine reinforcement, nicotine cue reactivity, and the potential effect of genetic polymorphisms on both of those phenotypic measures (*n* = 55; Protocol #10‐DA‐N456). The second study investigated the effects of gemfibrozil on the same measures (*n* = 24; Gendy et al., 2018). To avoid the confounding effects of the medication, the current report includes only data from the placebo condition of the study. The final study increased the population sample size examining the same measures (*n* = 25; REB# 134/2015). Each study conducted the same forced choice and cue‐reactivity sessions (see below) on regular smokers. Regular smokers were recruited from the different sites in both the United States and Canada. Participants were eligible if they were 18–64 years old, smoked at least 10 cigarettes per day (CPD) for at least 1 year, had positive urinary cotinine levels (for smoking confirmation), and were medically and psychologically healthy. To determine overall health, medical and psychiatric history was collected in all studies. Subjects were ineligible if they were seeking treatment for nicotine dependence, recently used nicotine replacement products, consumed more than 15 alcohol drinks per week, used illicit drugs regularly, were pregnant or nursing, or used medications that would be unsafe during experimental sessions. All studies were conducted according to the Declaration of Helsinki (7th revision) as well as protocols approved by the respective review boards of the National Institute of Drug Abuse (NIDA), Baltimore, Maryland, U.S. and the Centre for Addiction and Mental Health (CAMH), Toronto, Ontario, Canada.

### 2.2 Study design

Potential participants were first invited to an in‐person eligibility assessment. During this session, informed consent was obtained; then, demographic data and drug use history were collected. Breathalyzers were used to collect breath samples for breath alcohol concentration (BrAC) and carbon monoxide (CO) estimation. This information was used to verify positive smoking and negative drinking status. Urine was collected was screened for current drug use and pregnancy (for females only). Vital signs were collected, and participants completed several questionnaires (see below). Participants that met inclusion criteria were enrolled in the study, provided blood for genotyping, and scheduled to complete both forced choice and cue‐reactivity experimental sessions.

### 2.3 Genotyping

The CB1R (rs2023239 T>C) was determined using the Taqman SNP genotyping assay performed on a ViiA7 thermal cycler (Life Technologies) with appropriate controls. Briefly, 5 µl of 2× GTXpressTM Master mix (cat#4401890, Life Technologies) is mixed with 10 ng of DNA and the 40× probe (cat#4351379_10, Life Technologies) in a final volume of 10 µl and run for 50 cycles of 95°C for 1 s and 60°C for 20 s.

### 2.4 Questionnaires

The Fagerstrom Test for Nicotine Dependence (FTND; Heatherton et al., 1991) was used to capture severity of nicotine dependence during the eligibility assessment. During the forced choice session, the modified Cigarette Evaluation Questionnaire (mCEQ; Cappelleri et al., 2007) was used to measure the subjective effects of cigarettes. During the cue‐reactivity session, craving was assessed by Tobacco Craving Questionnaire—Short Form (TCQ‐SF; Heishman et al., 2008) and the Visual Analogue Scale (VAS; Weinberger et al., 2012). Using the VAS, participants were asked to rate how much they “craved” and “urged” for a cigarette and that specific moment. Mood was also measured during the cue‐reactivity session using the Mood Form (Diener & Emmons, 1984), and the VAS, where they were asked about “positive” and “negative” mood at that specific moment.

### 2.5 Experimental procedures

These procedures have been thoroughly described elsewhere (Chukwueke et al., 2020). Briefly, participants underwent a forced choice paradigm before the cue‐reactivity session. Each experimental session was conducted in a room that was ventilated to allow indoor smoking and was preceded by a smoking deprivation period where participants were asked to smoke four puffs of their own cigarette then relax for 30–60 min. This standardized the time since last cigarette. After this smoking deprivation period, the experimental session began.

#### 2.5.1 Forced choice

This double‐blinded procedure assessed the relative reinforcing effects of nicotine by comparing subjective ratings and puff choices between Nic and Denic cigarettes (De Wit & Johanson, 1987; Perkins et al., 1996). This session occurred in two phases where the participants started by an exposure phase in which they were able to sample the research cigarettes. These cigarettes differed in amounts of nicotine (see “cigarette” section). There were four exposure trials where each participant sampled either Nic (A) or Denic (B) color‐coded cigarettes in an ABAB or BABA counterbalanced order. Exposure trials were completed in 20–30 min intervals to simulate a regular smoking behavior (eight puffs every 40 min (Hatsukami et al., 1988). After each exposure trial, participants completed the mCEQ.

After the cigarette exposure phase (20–30 min later), participants completed the four trials of the forced choice task. In this task, participants were presented new Nic and Denic cigarettes concurrently. They were instructed to smoke a total of four puffs from either cigarette in any combination they pleased. Participant choice was recorded by observation from a control room containing a 2‐way mirror.

##### Cigarettes

Due to manufacturing and accessibility constraints, the cigarettes used in the various studies differed slightly. The Nic cigarettes used included Quest^®^ 1 cigarettes (Vector Tobacco; 0.6 mg nicotine yield), SPECTRUM^®^ research cigarettes (RTI international; 0.9 mg nicotine yield), and commercially available Players Rich brand cigarettes. Denic cigarettes included Quest^®^ 3 cigarettes (<0.05 mg nicotine yield) and SPECTRUM^®^ research cigarettes (0.03 mg nicotine yield).

#### 2.5.2 Cue reactivity

After the smoking deprivation period, participants provided a CO sample and completed baseline self‐report questionnaires (TCQ‐SF, VAS, and Mood Form). During this session, participants were presented with an opaque container that contained either a smoking related or neutral cue. All participants underwent each cue condition once, in a counter balanced manner. Participants were instructed to open the container and interact with the objects inside. The smoking cue condition included a container that housed a pack of commercially available cigarettes, a lighter, and an ash tray. Participants were told to light the cigarette without puffing and hold the cigarette for 30–60 s, after which it was extinguished. The neutral cue condition included a container that house a pack of pencils, a sharpener, and a note pad. Participants were instructed to take a pencil, sharpen it, and hold it as if writing for 30–60 s. Participants completed self‐report measures of craving and mood (TCQ‐SF, VAS, and Mood Form) prior to cue exposure (baseline) and 15 min after cue exposure. Physiological readings (heart rate, blood pressure, skin conductance, and skin temperature) were collected throughout the session.

### 2.6 Statistical analysis

Participants were grouped according to their genotype (CC + CT [C group] vs. TT genotypes [No C Group]), and analyses were conducted to explore the role of the CB1R rs2023239 genotypes in the forced choice and cue‐reactivity paradigms. For the forced choice session, subjective responses to cigarette types were evaluated using the mCEQ. Analysis included both the mCEQ composite score (Harrell et al., 2016) and subscales (psychological reward, aversion, craving reduction, respiratory tract sensation, and smoking satisfaction). The behavioral response to the forced choice examined number of puff choices on Nic versus Denic cigarettes (for a total of 16 forced choices). Both subjective and behavioral variables were analyzed using a repeated‐measures ANOVAs where cigarette type (Nic, Denic) was a within‐subject variable, while sex (male, female) and genotype group (C group, No C group) were between‐subjects variables.

Cue‐reactivity outcomes were collected at two time points (baseline and 15 min after cue). Values over time were calculated as difference scores (15 min after cue minus baseline). Difference scores of subjective (TCQ‐SF [general and individual factors], VAS [craving and mood], Mood Form) and physiological (skin conductance, skin temperature, heart rate, and blood pressure) data were calculated. These variables were analyzed using a repeated‐measures ANOVAs where cue type (Neutral, Smoking) was a within‐subject variable, sex (male, female), and genotype group (C group, No C group) were between‐subjects variables. As secondary analyses, the same model was used to analyze additional VAS outcome measures that were collected during cue presentation (difference scores; during cue presentation minus baseline).

To analyze the potential effect of the various confounding variables (cigarette brand, study), separate analyses were conducted. Identical statistical models as above were used with the addition of cigarette brand as a between subject variable in the forced choice analysis and race (white, black, and others) as a between subject variable in the data analysis for both forced choice and cue reactivity.

For all analyses, missing data were substituted with means, and results were considered significant at *p* < .05 (SPSS ver. 24.0/25.0). Bonferroni's corrections were applied on all multiple comparisons. Standardized effects sizes for the variance explained are reported as partial eta‐squared ($\eta_{p}^{2}$).

## 3 RESULTS

### 3.1 Demographics

A total of 104 participants were recruited, genotyped, and completed all components of the study (See Table 1). There were no differences between the two genotype groups across all demographic and smoking characteristics, except for race. In the C genotype group, there was a more even proportion of white (46%) and black (48%) participants compared to the No C genotype group which had a higher concentration of white participants (69%) (*p* = .004). Because of the genotype frequency differences among the races, race was included in the secondary analysis. Data analysis on forced choice data included the entire data set (*n* = 104) while analysis on cue reactivity (*n* = 103) excluded one participant due to a complete lack of cue‐reactivity questionnaire data.

**TABLE 1:** This table describes the demographic and smoking characteristics of our study sample

	Total *N* = 104	C allele group (CC + CT) *n* = 39	No C allele group (TT) *n* = 65	2‐tailed *t* test and chi‐square analysis (C vs. No C group)
	*n* (%)	*n* (%)	*n* (%)	*p*
Demographic characteristics				
Sex				
Male	57 (54.8)	21 (53.8)	36 (55.4)	n.s.
Female	47 (45.2)	18 (46.2)	29 (44.6)	
Race				
Black	31 (29.8)	19 (48.7)	12 (18.5)	.004
White/Caucasian	63 (60.6)	18 (46.2)	45 (69.2)	
Other	10 (9.6)	2 (5.1)	8 (12.3)	
Education				
> high school	63 (60.6)	20 (51.3)	43 (66.2)	n.s
	***M* ± *SD***	***M* ± *SD***	***M* ± *SD***	***p***
Age (years)	41.80 ± 11.07	41.74 ± 11.37	41.83 ± 10.98	n.s.
Smoking characteristics				
Cigarettes per day (CPD)	17.48 ± 5.85	17.74 ± 6.69	17.32 ± 5.34	n.s.
No. of smoking years	22.27 ± 11.42	21.26 ± 10.5	22.88 ± 11.99	n.s.
Pack‐years	19.90 ± 12.81	18.29 ± 10.27	20.87 ± 14.11	n.s.
Fagerstrom Test for Nicotine Dependence (FTND)	5.35 ± 1.89	5.44 ± 1.89	5.30 ± 1.91	n.s.

Note

The sample was compared across genotype groups using chi‐square analysis for categorical variables and 2‐tailed *t* tests for continuous variables. Note, the “other” race category includes Asian, Latin, American Native, and mixed ethnicities.

Abbreviation: n.s., not significant.

### 3.2 Nicotine reinforcement

#### 3.2.1 Effects of genotype on subjective and behavioral measures

**FIGURE 1:**
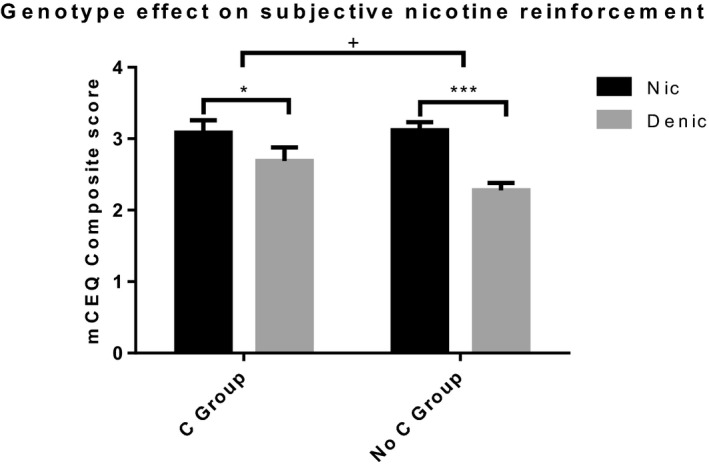
mCEQ composite score for Nic and Denic cigarettes across both genotype groups. There is a 2‐way cigarette type × genotype variant interaction (*F* (1,100) = 4.588, *p* = .040, $\eta_{p}^{2}$ = 0.042). For both genotype groups, the Nic cigarette elicited significantly greater mCEQ composite scores than the Denic cigarette. However, this effect was smaller in the C group compared to the No C group. Denic, Denicotinized; mCEQ, Modified Cigarette Evaluation Questionnaire; Nic, Nicotinized. + = significant 2‐way interaction. * = *p* < .05. *** = *p* < .001

There was a genotype × cigarette type interaction observed in the mCEQ composite score (Figure 1; *F* (1,100) = 4.336, *p* = .040, $\eta_{p}^{2}$ = 0.042). Bonferroni corrected multiple comparisons revealed that Nic cigarettes were rated higher than Denic cigarettes in both genotype groups. However, this effect was smaller in the C group (Nic‐Denic mean difference = 0.42, *p* = .009) compared to the No C (Nic‐Denic mean difference = 0.84, *p* < .001) group, suggesting a lower subjective reward effect in the C group. Analyses on the mCEQ subscales found similar results in the “smoking satisfactions” (*F* (1,100) = 3.981, *p* = .049, $\eta_{p}^{2}$ = 0.038) and “enjoyment of the respiratory tract sensation” (*F* (1,100) = 6.893, *p* = .010, $\eta_{p}^{2}$ = 0.064) subscales (Figure S1). In these subscales, there was a genotype × cigarette type interaction, where Nic ratings relative to Denic ratings were lower for the C group compared to the No C group. This also suggests lower subjective effects in those with the C genotype. This may indicate that the decreased subjective reward effect seen in the composite scores among those with the C genotype may be due to a reduction in the enjoyment of smoking and/or the resulting respiratory sensation.

**FIGURE 2:**
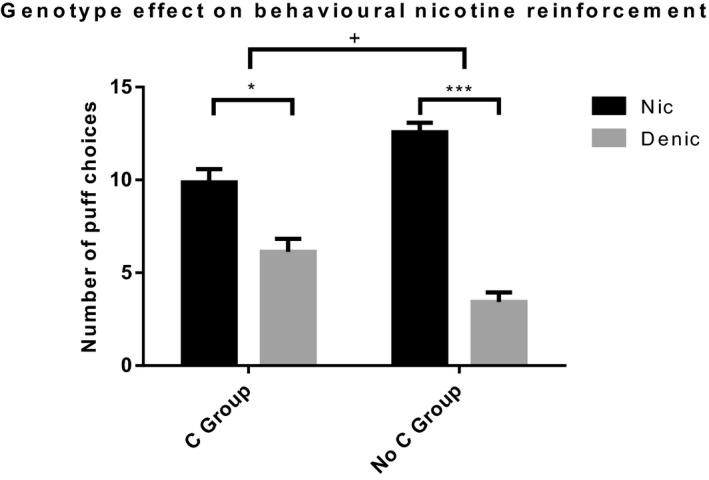
Number of puff choices from Nic and Denic cigarettes across both genotype groups. There was a cigarette × genotype interaction (*F* (1,100) = 9.919, *p* = .002, $\eta_{p}^{2}$ = 0.090). In both genotype groups, there were significantly more Nic puffs than Denic puffs. However, this effect was smaller in the C group compared to the No C group. Denic, Denicotinized; Nic, Nicotinized. + = significant 2‐way interaction. * = *p* < .05. *** = *p* < .001

In the behavioral responses of the forced choice task, there was a genotype × cigarette type interaction (Figure 2; *F* (1,100) = 9.919, *p* = .002, $\eta_{p}^{2}$ = 0.090). Bonferroni corrected multiple comparisons revealed that Nic puffs were chosen more than Denic puffs in both groups. However, similarly to the subjective measures, this effect was smaller in the C group (Nic‐Denic mean difference = 3.73, *p* = .008) compared to the No C group (Nic‐Denic mean difference = 9.21, *p* < .001). Consistent with the subjective results, this suggests decreased nicotine reinforcement in the C group as assessed by behavioral responses in our forced choice task.

#### 3.2.2 Controlling for race and cigarette brand

There were no changes in the pattern of results when cigarette brand entered the model. While controlling for race, the significant genotype × cigarette effects seen in the mCEQ scores were not maintained. However, the genotype effect on the behavioral forced choice task was maintained (*F* (1,97) = 5.600, *p* = .020, $\eta_{p}^{2}$ = 0.055). This suggests that the C group experiences a lower nicotine reinforcing effect as measured by the behavioral forced choice task even when accounting for the influence of race and cigarette brand (see Figure S5–S34 for separate analysis by racial group).

#### 3.2.3 Main effect of cigarette

Main effects of cigarette type were revealed in the mCEQ composite score (*F* (1,100) = 39.867, *p* < .001, $\eta_{p}^{2}$ = 0.285) and all subscales (“smoking satisfaction” (*F* (1,100) = 40.653, *p* < .001, $\eta_{p}^{2}$ = 0.289); “psychological reward” (*F* (1,100) = 22.705, *p* < .001, $\eta_{p}^{2}$ = 0.185); “enjoyment of the respiratory tract sensation” (*F* (1,100) = 17.304, *p* < .001, $\eta_{p}^{2}$ = 0.148); “craving reduction” (*F* (1,100) = 41.946, *p* < .001, $\eta_{p}^{2}$ = 0.296)) with the exception of “Aversion.” As we have previously reported (Chukwueke et al., 2020), these results suggest that Nic cigarettes are generally rated higher than Denic cigarettes on subjective measures irrespective of genotype group.

In a similar vein, there was a main effect of cigarette type in the behavioral forced choice task (*F* (1,100) = 55.325, *p* < .001, $\eta_{p}^{2}$ = 0.356). In the behavioral forced choice task, Nic puffs (11.56 ± 4.42) were chosen more than Denic puffs (4.44 ± 4.42). Taken together, both the subjective and behavioral measures suggest that Nicotine containing cigarettes are more reinforcing than cigarettes without nicotine.

### 3.3 Cue reactivity

#### 3.3.1 Effect of genotype on cue‐reactivity measures

There were no effects of genotype group on any of our cue‐reactivity measures (craving, mood, and physiological readings) (Figure S2–S4). There were no genotype effects seen on the VAS outcome measures collected during cue presentation.

#### 3.3.2 Main effect of cue

There were main effects of cue on both positive (*F* (1,99) = 3.983, *p* = .049, $\eta_{p}^{2}$ = 0.039) and negative mood (*F* (1,99) = 7.086, *p* = .009, $\eta_{p}^{2}$ = 0.067) measures assessed by the Mood Form (Figure S3). Smoking cues elicited greater decreases in positive mood, and greater increases in negative mood, compared to neutral cues. There were also main effects of cue on skin temperature (*F* (1,72) = 5.205, *p* = .025, $\eta_{p}^{2}$ = 0.067) and heart rate (*F* (1,57) = 4.609, *p* = .036, $\eta_{p}^{2}$ = 0.075) (Figure S4). Smoking cues elicited greater decreases in skin temperature and heart rate compared to neutral cues. These results suggest that smoking‐related environmental cues play a role in eliciting greater mood, temperature, and heart rate changes in regular smokers compared to neutral cues.

In analysis of VAS measures during cue presentation, there was an effect of cue type where smoking cues elicited greater craving than neutral cues on both the VAS‐crave (*F* (1,99) = 11.56, *p* = .001, $\eta_{p}^{2}$ = 0.105) and VAS‐Urge (*F* (1,99) = 18.38, *p* < .001, $\eta_{p}^{2}$ = 0.157) questions. There were no main effects on VAS‐mood measures.

### 3.4 Sex‐related effects

There were no effects of sex on neither the forced choice nor the cue‐reactivity procedures.

## 4 DISCUSSION

This study found Nic cigarettes elicited greater behavioral and subjective responses than Denic cigarettes and that this effect varied across the CB1R genotype groups. Specifically, smokers with at least one copy of the C allele variant of the CB1R rs2023239 SNP experienced a lower nicotine reinforcement effect compared to smokers without the C allele. Given that we hypothesized a positive relationship between the existence of the C allele and increased nicotine reinforcement and cue reactivity, these results did not support our hypothesis. Our results showed no interaction between the CB1R rs2023239 genotype group and our cue‐reactivity measures, also contrary to our hypothesis. However, there were main effects of cue type on certain physiological measures (skin temperature and heart rate), mood self‐reports (positive and negative mood), and transient main effects of cue type on craving (during cue presentation; VAS‐crave and VAS‐urge). Finally, contrary to our hypothesis, there was a lack of sex effects in our analyses.

The results from this study suggest that the CB1R rs2023239 SNP plays a role in nicotine reinforcement. Our findings of reduced subjective reward and behavioral nicotine reinforcement effects in those with the C allele (CC or CT genotype) are difficult to interpret considering previous reports. Previous research has shown that the level of CB1R function corresponds with nicotine reinforcement. Specifically, animal studies have shown that pharmacological blockade of CB1Rs decreases nicotine self‐administration (Cohen et al., 2002) while stimulation of CB1R increases nicotine intake (Gamaleddin et al., 2012). This line of research suggests that an increase or decrease in CB1R functioning would correspond to a similar change in nicotine reinforcement. Moreover, studies showed that individuals with the CB1R rs2023239 C allele would have increased CB1R expression (Hirvonen et al., 2013; Hutchison et al., 2008), presumably resulting in increased CB1R functioning. Together, these findings would suggest that an increase in CB1R levels would correspond to an increase in nicotine reinforcement. However, in our study, individuals with this allele experienced a lower nicotine reinforcement effect, which is inconsistent with previous animal research.

This inconsistency may be due to several different factors. First, there are methodological discrepancies in the body of research using difference in test species (animal vs. human) and laboratory measures (self‐administration/CPP vs. forced choice). Second, the magnitude of effect of the rs2023239 SNP on CB1R levels still needs to be fully verified. An unclear understanding of the variation effect may contribute to inconsistent findings. Finally, our sample includes only current smokers; participants who have self‐selected to be sensitive to the rewarding properties of nicotine. Nonetheless, our results add to the scientific literature by providing evidence in support of an impact of CB1R rs2023239 variation in subjective reward and behavioral nicotine reinforcement, as assessed by the forced choice paradigm.

The results from our cue‐reactivity procedure suggest that the CB1R rs2023239 C allele may not play a role in tobacco cue‐induced craving or mood and physiological changes. Contrary to our hypothesis, there was no genotype group difference on any of our cue‐reactivity outcome measures. Previous animal research has shown that selective blockade of CB1Rs reduces the reinstatement of previously extinguished nicotine‐seeking behavior elicited by nicotine‐associated stimuli (Diergaarde et al., 2008; Forget et al., 2009). This suggests a role of CB1R in nicotine‐related cue‐induced seeking behavior in animals. However, similar findings have yet to be translated in human smokers. This may in part be due to heterogeneity in reactivity to environmental cues within the smoking population. Previous research has shown that different drugs reduce smoking cue reactivity via distinct mechanism of actions. For example, in smokers, varenicline treatment reduced cue reactivity via increased activation in the orbital frontal cortex (Franklin et al., 2011), whereas baclofen reduced cue reactivity through decreased avInsula activation (Ketcherside et al., 2020). This has led some authors to speculate the potential for subgroups within smokers with varying vulnerabilities, whereby varenicline may be more effective in abstinence initiation for smokers susceptible to avoidance of withdrawal, while baclofen promotes relapse prevention in smokers who are cue‐vulnerable (Ketcherside et al., 2020).

Furthermore, the rs2023239 C allele has been implicated in craving induced by other substances of abuse. Human laboratory studies have shown that those with the rs2023239 C containing genotypes experience greater craving in response to cannabis (Haughey et al., 2008) and alcohol (Hutchison et al., 2008) related cues. These results support the functional role of the rs2023239 SNP in drug‐related craving. Yet considering our findings, the role of rs2023239 C allele on cue‐induced craving may not extend to tobacco‐related cues in humans.

The current study did not find any sex‐related effects in either forced choice or the cue‐reactivity procedure. While previous research has shown a decrease in CB1R expression in male smokers that study did not compare males to female smokers (Hirvonen et al., 2018). At this point, there are limited studies investigating sex differences in the context of the CB1 system and nicotine dependence.

The present study is not without its limitations. First, while the rs2023239 C allele has been associated with increased CB1R expression (Hirvonen et al., 2013; Hutchison et al., 2008), a definitive role for a functional effect of the SNP is required. Second, our analyses grouped those homozygous for the minor C allele (CC genotype) and those heterozygous together (CC + CT), which was then compared to the homozygous major allele (TT). This gave us greater statistical power but reduced our understanding of the contributions of each genotype toward the overall findings. Third, there were some inconsistencies in the study procedures between the different studies that were combined and reported here (Chukwueke et al., 2020). For example, one study employed a 30‐min deprivation period, while another used a 60‐min period. It is unclear how differences in these procedures affected the results. Finally, the study was not designed to adequately account for the effect of race. While our behavioral measures of reinforcement were maintained, our subjective results did not withstand controlling for race. We were underpowered to delineate the contribution of race in our analyses as our population sampling was not designed to consider race. Analyzing the races separately, due to the potential for ancestry differences in the genomic impact of the CB1R rs2023239 SNP, indicated a similar pattern of results as was observed in the collapsed analysis. While the topic of racial contribution in research is contended and its utility in scientific examination is complex (Keita et al., 2004), the issue of race in the context of nicotine reinforcement and cue‐elicited craving warrants further investigation. Future studies should include larger sample sizes, consider race with sampling the population, and maintain greater consistency within/between clinical studies to address these limitations.

In conclusion, our study shows that the rs2023239 C containing genotypes may alter nicotine reinforcement but may have limited involvement in tobacco smoking cue‐elicited craving. Specifically, smokers with the C allele (CC + CT genotype) experienced a decreased effect, compared to smokers without this allele, in our measure of nicotine reinforcement. On the other hand, we found no CB1R rs2023239 SNP effect in our cue‐reactivity paradigm. These intriguing findings suggesting a role for CB1R variation in nicotine reinforcement require validation and a better understanding of the mechanistic properties at play.

## CONFLICT OF INTEREST

RF Tyndale has consulted for Quinn Emanuel and Ethismos Research Inc.

## AUTHOR CONTRIBUTION

CC and WK contributed equally to the subject recruitment, data analysis, and manuscript preparation. RT, MG, and RT provided methodological oversight and helped with review/editing. SH and BLF secured funding and provided supervision over the methodology, data analysis, and manuscript preparation.

### Peer Review

The peer review history for this article is available at https://publons.com/publon/10.1002/brb3.1982.

## Supporting information

Figure S1‐S34

## Data Availability

All additional data, research protocols, and information on materials used in this investigation will be made readily available upon request as allowed by the governing review boards of the involved research institutions.
