# Supplementary material for: The CB1R rs2023239 receptor gene variant significantly affects the reinforcing effects of nicotine, but not cue reactivity, in human smokers
Source: Brain Behav. 2020 Dec 25;11(2):e01982. doi: 10.1002/brb3.1982 (PMC7882168; doi:10.1002/brb3.1982)
Supplement: Supplementary file 1 — Figure S1‐S34 [file BRB3-11-e01982-s001.docx]

**Supplemental Graphs**

**Supplemental Figure 1.**

**mCEQ Subscales**

**Legends:** mCEQ scores of individual subscales. There were genotype × cigarette type interactions in both the ‘smoking satisfaction’ (F (1,100) = 3.981, p = 0.049, η^2^_p_ = 0.038) and ‘enjoyment of the respiratory tract sensation’ (F (1,100) = 6.893, p = 0.010, η^2^_p_ = 0.064). In both subscales, the Nic-Denic mean difference was smaller in the C group compared to the No C group. There was a main effect of cigarette type in all subscales, where Nic scores were significantly greater than Denic scores, except for Aversion. + = significant 2-way interaction. * = p < 0.05. *** = p < 0.001

**Supplemental Figure 2.**

Legend: There were no genotype × cigarette type interaction in any of the craving measures. There was also no main effect of cue type in any measure.

**Supplemental Figure 3.**

Legend: There were main effects of cue type in both positive mood (F (1,99) = 3.983, p = 0.049, η^2^_p_ = 0.039) and negative mood (F (1,99) = 7.086, p = 0.009, η^2^_p_ = 0.067). The smoking cue elicited significantly greater decreases in positive mood and significantly greater increases in negative mood compared to the neutral cue.

**Supplemental Figure 4.**

Legend: There were main effects of cue type in both heart rate (F (1,57) = 4.609, p = 0.036, η^2^_p_ = 0.075) and skin temperature (F (1,72) = 5.205, p = 0.025, η^2^_p_ = 0.067). The smoking cue elicited significantly greater decreases in heart rate and skin temperature compared to the neutral cue.

**Supplemental Graphs- Race analysis**

| Forced Choice | | |
| --- | --- | --- |
| All (Race collapsed) | White | Black |
| 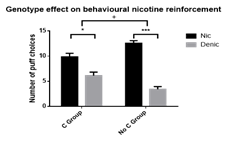 | 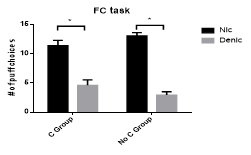 | 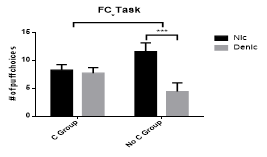 |
| Supp. Fig. 5 | Supp. Fig. 6 | Supp. Fig. 7 |

| mCEQ Composite score | | |
| --- | --- | --- |
| All (Race collapsed) | White | Black |
| 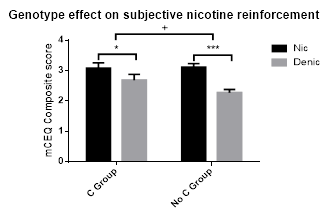 | 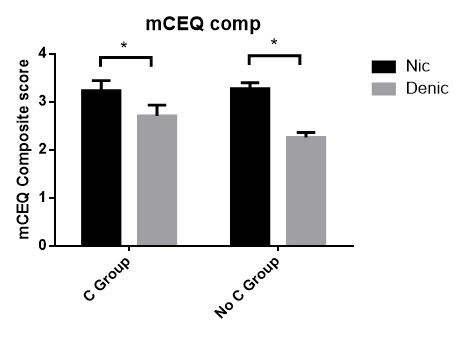 | 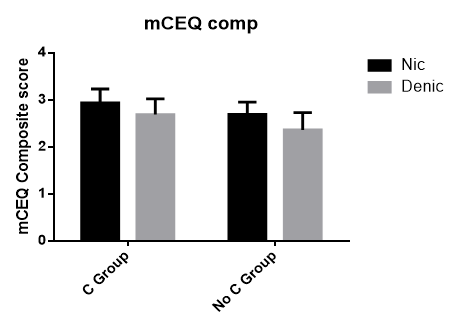 |
| Supp. Fig. 8 | Supp. Fig. 9 | Supp. Fig. 10 |

| mCEQ Subscales | | | | |
| --- | --- | --- | --- | --- |
| All (Race collapsed) | White | | Black | |
| 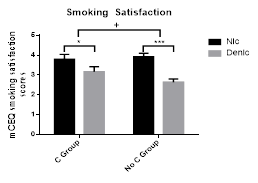 | 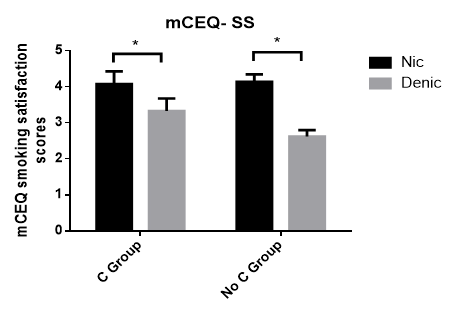 | | 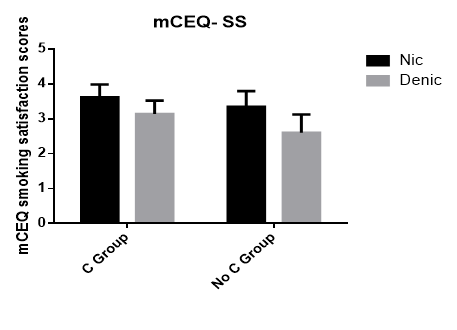 | |
| Supp. Fig. 11 | Supp. Fig. 12 | | Supp. Fig. 13 | |
| 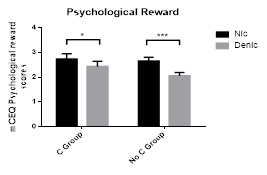 | 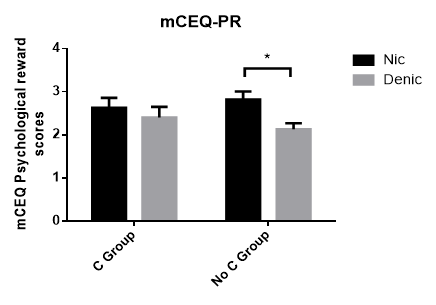 | | 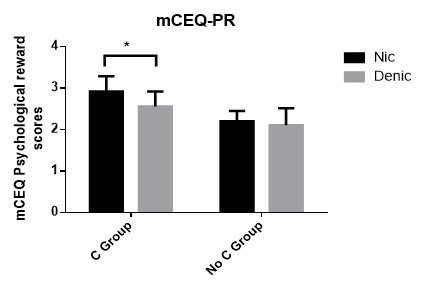 | |
| Supp. Fig. 14 | Supp. Fig. 15 | | Supp. Fig. 16 | |
| 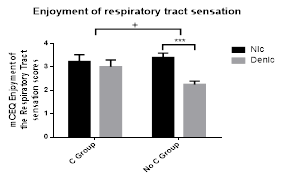 | 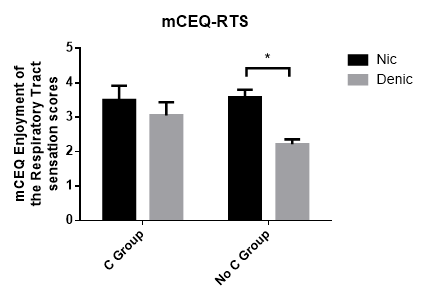 | | 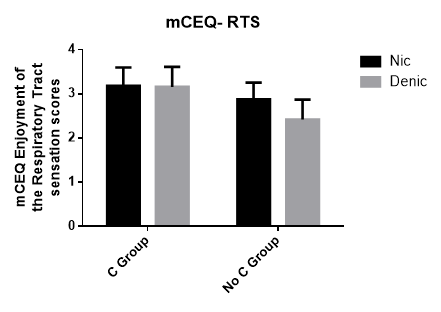 | |
| Supp. Fig. 17 | Supp. Fig. 18 | | Supp. Fig. 19 | |
| 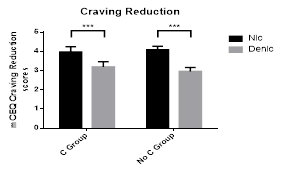 | 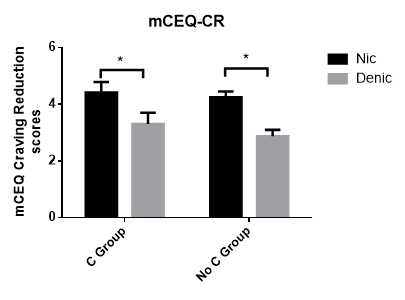 | | 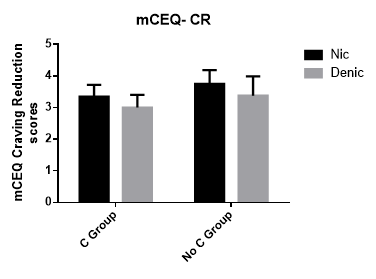 | |
| Supp. Fig. 20 | Supp. Fig. 21 | | Supp. Fig. 22 | |
| 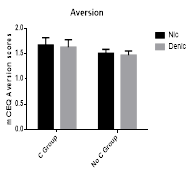 | 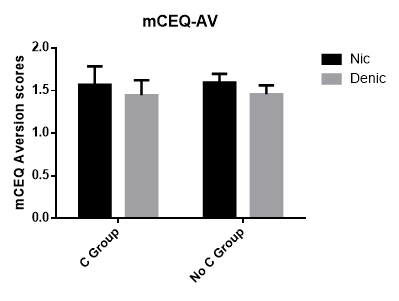 | | 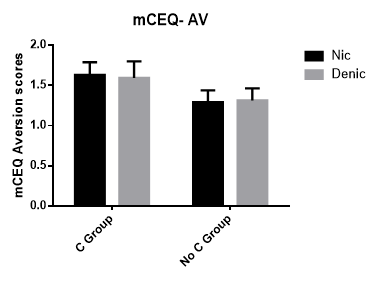 | |
| Supp. Fig. 23 | Supp. Fig. 24 | | Supp. Fig. 25 | |
| Cue-reactivity (Craving) | | | | |
| All (Race collapsed) | | White | | Black |
| 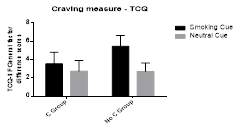 | | 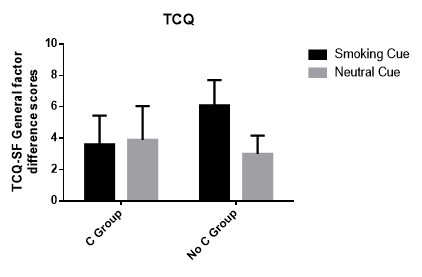 | | 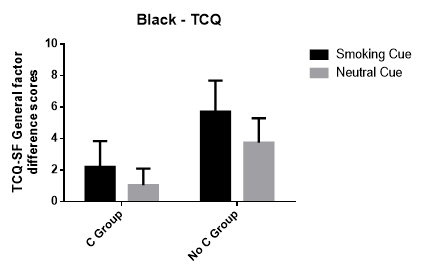 |
| Supp. Fig. 26 | | Supp. Fig. 27 | | Supp. Fig. 28 |
| 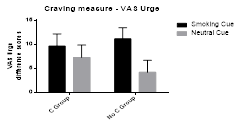 | | 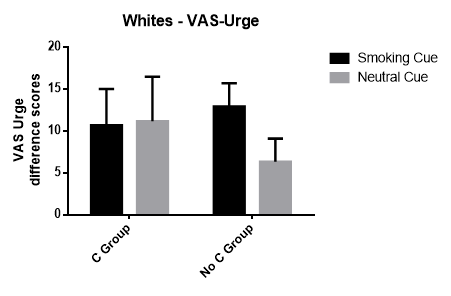 | | 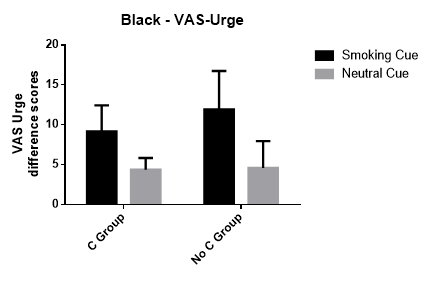 |
| Supp. Fig. 29 | | Supp. Fig. 30 | | Supp. 31 |
| 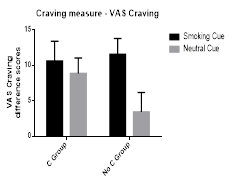 | | 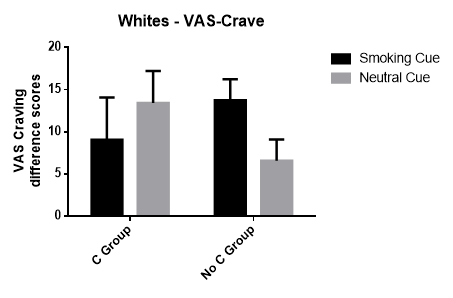 | | 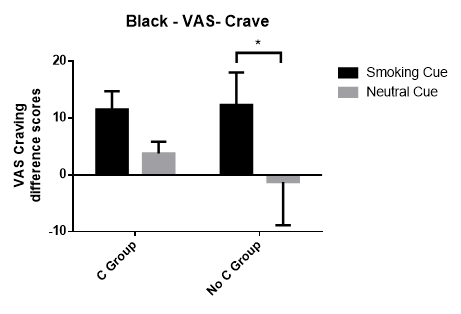 |
| Supp. Fig. 32 | | Supp. Fig. 33 | | Supp. Fig. 34 |
